# Editorial: Molecular drivers of immunothrombosis

**DOI:** 10.3389/fimmu.2024.1385966

**Published:** 2024-02-27

**Authors:** Alice Assinger, Madhumita Chatterjee, James D. McFadyen

**Affiliations:** ^1^ Institute of Vascular Biology and Thrombosis Research, Center of Physiology and Pharmacology, Medical University of Vienna, Vienna, Austria; ^2^ Department of Pharmacology, Experimental Therapy and Toxicology, University Hospital Tübingen, Tübingen, Germany; ^3^ Atherothrombosis and Vascular Biology Laboratory, Baker Heart and Diabetes Institute, Melbourne, VIC, Australia

**Keywords:** immunothrombosis, platelets, infection, inflammation, immune response

Inflammation and thrombosis are highly evolutionary conserved and intimately linked biological processes. Indeed, it is now apparent that the inflammatory response can initiate thrombosis, the corollary being that the haemostatic response plays an important role in preventing the dissemination of invading organisms. The phenomenon of immunothrombosis, where thrombosis is initiated by inflammatory responses, has been most widely studied in sepsis, however it is now evident that immunothombosis play an important role in a range of important clinical conditions including COVID-19, ischaemia reperfusion injury and venous thromboembolic disease. As such, immunothrombosis represents a double-edged sword - on the one hand immunothrombotic processes likely serve an important defense mechanism to invading pathogens, however, on the other hand when dysregulated can lead to macro- and microvascular thrombosis and organ damage. Whilst it is well appreciated that platelets, leukocytes and the endothelium all play an important role in immunothrombosis, the precise molecular mechanisms that regulate immunothrombosis remain ill defined. This is critical since, to date, there are no effective therapies to treat immunothrombosis, thus understanding the precise mechanisms underpinning immunothrombosis is critical to develop therapeutic approaches.

Through this prism, in this Research Topic, Maneta et al. provide a comprehensive review of the current knowledge on endothelial dysfunction and immunothrombotic processes in sepsis. This provides new insights to our understanding of how endothelial dysfunction and microthrombotic complications can ultimately lead to organ damage and mortality in this complex condition. The authors discuss organ specific mechanisms of endothelial dysfunction and summarize therapeutic strategies, highlighting recent advances in interventional approaches that may improve outcome in septic patients.

Cox provides another perspective by placing a spotlight on the role of platelets as central actors in mediating septic complications. Sepsis is accompanied by thrombocytopenia which confers a poor prognosis in many infectious diseases. Thrombocytopenia along with disseminated intravascular coagulopathy not only increases the thrombotic risk but also the bleeding risk in this complex disease. Although historically considered mere bystanders in sepsis, a large body of data now demonstrates that platelet have important immune functions. Accordingly, many pathogens can directly interact and activate platelets via platelet expressed adhesion and immune receptors. This review provides a detailed discussion of the role of platelets in sepsis and the potential beneficial role of anti-platelet agents to inhibit specific platelet-pathogen interactions, and in managing septic complications. The important perspective is that novel strategies are necessary to allow for inhibition of platelet functions relevant to sepsis and platelet pathogen interactions without increasing the bleeding risk in these vulnerable patients. Thrombocytopenia is not only commonly observed in patients with sepsis but also in patients with severe COVID-19. Cheng et al. provides an overview of the mechanisms of thrombocytopenia in sepsis and a contemporary perspective regarding how COVID-19 infection may cause thrombocytopenia. The authors also discuss recent findings on how preventing thrombocytopenia may improve outcomes in both sepsis and COVID-19 patients.

The need for novel therapeutic strategies was highlighted during the COVID-19 pandemic. In a signaling network analysis of platelet proteomes from patients with SARS-CoV-2 Osmanoglu et al. highlight fostamatinib as a drug candidate to control platelet hyperactivation and thrombotic events during COVID-19. These data were generated from platelet proteomes from patients exposed to the ancestral strain of SARS-CoV-2. However, much interest has focused on whether COVID variants elicit differential biological responses. In this context, the study by Garcia et al. demonstrated that whilst SARS-CoV-2 Omicron has different biological characteristics compared to prior variants, platelet activation and desensitization is quite similar to the Delta variant. Omicron induced selective autophagy in patients with severe disease, but the mechanisms of intraplatelet processing of Omicron cargo, as part of the innate response, differs from Delta, suggesting that mutations on spike protein modify virus interactions with platelets.

Infections and inflammatory diseases lead to the activation of the immune system and release of a plethora of chemokines, which apart from modulating immune responses also has an impact on the haemostatic system. In their comprehensive review, Leberzammer and von Hundelshausen unravel the role of chemokines in thrombosis from a cellular perspective intricately dissecting the diverse mechanisms through which chemokines influence thrombotic processes. Considering endothelial cells, platelets and leukocytes in an interactive network, the authors shed light on the complex mechanisms employed by chemokines including engagement of cognate receptors, receptor-independent processes, involvement of atypical receptors, signalling complexes, alteration of cell surface charge, interaction with coagulation factors, and activation of platelet receptors, to foster aggregation, thrombosis and thrombo-inflammation. In a clinical study Sopova et al. demonstrate the prognostic significance of the chemokine interferon-gamma inducible protein 10 (IP-10) post-myocardial infarction. They found that increased serum levels of IP-10 in the acute phase of STEMI predict a better recovery in cardiac systolic function and less adverse events in patients after STEMI, emphasizing the influence of chemokines in haemostasis and repair processes. Beyond chemokines, acute-phase proteins, particularly C-reactive protein (CRP), bridge the realms of thrombosis and hemostasis. Dix et al. delve into the intricate nature of CRP, shedding light on its pro-inflammatory and pro-thrombotic properties. The destabilized isoforms of CRP emerge as central players in atherothrombosis and venous thromboembolism, directly activating platelets and triggering the classical complement pathway. This exploration adds layers to our understanding of the interplay between inflammation and thrombosis, opening potential avenues for therapeutic interventions.

Platelets do not only contribute to thrombotic processes in cardiovascular diseases but also foster inflammation. The state of heightened inflammation resulting from thrombotic processes and vice versa is termed thrombo-inflammation. Reusswig et al., have shown that platelets become activated and migrate into the infarct zone, after acute myocardial infarction, where they trigger acute inflammation and cardiac remodeling, leading to alterations in scar formation and cardiac function. They decipher a crucial involvement of GPVI, the major collagen receptor in platelets, in this context. While GPVI could not intrinsically be linked to inflammatory responses, the authors demonstrate that collagen and thrombin signaling enhance pro-coagulant activity and worsen cardiac remodeling. These findings might pave the way for novel treatment options in these patients. This is particularly relevant given GPVI inhibitors have entered clinical trials.

While the influence of inflammatory mediators on the haemostatic system has been acknowledged for quite some time, the role of neuronal mediators remains less understood. Neuronal guidance proteins (NGPs) constitute a diverse group of proteins responsible for guiding neuronal axon navigation and refining neuronal circuits. Emerging evidence suggests that NGPs also play crucial immunomodulatory roles and impact platelet function. In an extensive review, Tang et al. elucidate the contributions of various NGPs, including semaphorins, plexins, and ephrins, to platelet formation and activation. Körner et al. narrow their focus to a specific NGP, endothelial Semaphorin 7A (SEMA7A), which exhibits both pro- and anti-inflammatory actions. The authors demonstrate that SEMA7A’s function is contingent upon the expression of target receptors within the affected organ systems. While SEMA7A exacerbates inflammation during tissue hypoxia, it induces the production of the anti-inflammatory interleukin 10 in a model of inflammatory peritonitis. Another understudied molecule with crucial functions as a molecular driver in inflammation and thrombosis is zinc (Zn2+). Despite its importance, our knowledge of the transport mechanisms that govern Zn2+ homeostasis in platelets is currently limited. The study by Amro Elgheznawy et al. addresses this gap by highlighting ZIP1 and ZIP3 as key regulators essential for maintaining both platelet Zn2+ balance and function.

Deciphering the underlying mechanisms that govern immunothrombotic and thrombo-inflammatory processes represents a prerequisite for novel therapeutic interventions. Discovering innovative antiplatelet therapies holds the potential to not only enhance the treatment of infections and address subsequent immunothrombotic complications but also offers advantages in the realm of cancer prevention and treatment. In Jessie Zhao et al.‘s review, an overview is presented on how platelets contribute to the cancer landscape and why they represent valuable targets for the development of novel tools and therapeutics in cancer diagnosis.

In their study Yan et al., sheds new light on the association between the abacavir based combined antiretroviral therapy (cART), and the increased risk of cardiovascular events. Here, the authors demonstrate that patients with HIV infection treated with abacavir based cART exhibit increased thrombin generation in a process linked to enhanced prothbombin conversion, thereby potentially providing an important clue regarding the mechanism by which abacavir may be linked to an increased rate of thrombotic events.

Collectively, this Research Topic provides a comprehensive overview, and novel insights, regarding the intricacies and complexity of immunothrombosis. In particular, it further serves to highlight the broad range of important clinical conditions where immunothrombosis plays an important role. Thus, we hope this spurs further research in unravelling the molecular drivers of immunothrombosis, which will hopefully ultimately yield innovative therapeutics for the treatment of the broad range of pathologies associated with immunothrombosis.

## Author contributions

AA: Funding acquisition, Writing – original draft, Writing – review & editing. MC: Writing – original draft, Writing – review & editing, Funding acquisition. JM: Writing – original draft, Writing – review & editing, Funding acquisition.

